# Effect of Kinematic Chain Exercise Protocol on Throwing Performance and Shoulder Muscle Strength among University Shot Put Athletes—A Randomized Controlled Trial

**DOI:** 10.3390/jcm13174993

**Published:** 2024-08-23

**Authors:** Vinod Kumar Kanakapura Chananke Gowda, Shenbaga Sundaram Subramanian, Riziq Allah Mustafa Gaowgzeh, Samira Ahmed Alsenany, Sally Mohammed Farghaly Abdelaliem, Amany Anwar Saeed Alabdullah, Alkhateeb M. Afnan

**Affiliations:** 1 College of Physiotherapy, Dayananda Sagar University, Devarakaggalahalli, Harohalli Kanakapura Road, Dt, Ramanagara 562112, Karnataka, India; vinod-mpt@dsu.edu.in; 2 Saveetha College of Physiotherapy, Saveetha Institute of Medical and Technical Sciences (SIMATS), Chennai 600077, TamilNadu, India; 3 Department of Physical Therapy, Faculty of Medical Rehabilitation Sciences, King Abdulaziz University, Jeddah 21589, Saudi Arabia; rizikjoresearch@mail.com (R.A.M.G.); amalkhateeb@kau.edu.sa (A.M.A.); 4 Department of Public Health, Faculty of Nursing, King Abdulaziz University, Jeddah 21589, Saudi Arabia; salsenany@kau.edu.sa; 5 Department of Nursing Management and Education, College of Nursing, Princess Nourah bint Abdulrahman University, P.O. Box 84428, Riyadh 11671, Saudi Arabia; smfarghaly@pnu.edu.sa; 6 Department of Maternity and Child Health Nursing, College of Nursing, Princess Nourah bint Abdulrahman University, P.O. Box 84428, Riyadh 11671, Saudi Arabia; aaalabdullah@pnu.edu.sa

**Keywords:** shot put, kinematic chain, exercise regimen, shoulder muscle strength, throwing efficiency, performance improvement, training program, athlete satisfaction

## Abstract

**Background/Objectives**: This study looks at how a kinematic chain exercise regimen that targets the lower, core, and upper body affects university shot put participants’ shoulder muscle strength and throwing efficiency. This study fills an apparent research void on shot put training approaches by presenting a comprehensive kinematic chain workout program. It was anticipated that this method would improve performance the most, considering the complex biomechanical requirements of the sport. **Methods**: Eighty athletes aged (19.87 ± 1.31 years), were assigned into two groups at random: experimental (n = 40) and control (n = 40). While the control group carried on with their usual training, the experimental group participated in an 8-week kinematic chain training program. Pre- and post-training evaluations were carried out to evaluate shot put-throwing ability, shoulder muscle strength, and participant satisfaction with the exercise regimen. **Results**: The analyses were performed to evaluate the between- and within-group effects in the 10-week intervention period using a two-way ANOVA. This study demonstrated that, when compared to the control group, the athletes in the kinematic chain program had significantly increased throwing distance (*p* = 0.01) and shoulder muscle strength (*p* = 0.01). Furthermore, there was a significant increase (*p* = 0.005) in the athletes’ satisfaction levels with the workout program among those in the experimental group. **Conclusions**: In shot put athletes, this study suggests that a kinematic chain-focused strategy can improve throwing performance and shoulder muscle strength. The findings suggest that incorporating kinematic chain workouts into shot put training programs could be beneficial. However, conclusions should be drawn with caution, and further research is necessary to confirm the effectiveness of kinematic chain-based approaches across various sports and to understand their broader implications in sports science.

## 1. Introduction

Shot put athletes must possess strength, speed, and technique to execute well. Biomechanics and training techniques are essential for effective performance. To ensure the best possible distances and greatest force, the shot put emphasizes an athlete’s momentum and skill [1].

The shot put throwing technique involves a combination of linear and rotational movements, including the glide or rotational technique, which involves executing the shot at the optimal angle and velocity. With the glide technique, body weight is shifted linearly over the throwing circle, and the shot is released with force. On the other hand, the athlete spins inside the circle before releasing the shot using the rotating technique. Every style has unique biomechanical characteristics that require specific training to become proficient. Throwing practice allows athletes to apply learned skills under competitive conditions, enhancing their overall strength and speed [2].

Functional exercises and kinematic chain training have gained popularity in recent years, enhancing athletic performance through integrated exercises focusing on coordinated muscle recruitment for optimal movement efficiency [3]. Kinematic chain activation exercises improve movement patterns in throwing sports by engaging multiple muscle groups at the same time, promoting synchronicity and efficiency. This promotes proper sequencing, enabling greater force and velocity during the throwing motion. Throwing requires the coordinated activation of muscles throughout the body, from the lower body for power generation to the core for stability and energy transfer [4]. Core stability exercises strengthen the abdominal, lower back, and pelvis muscles, enhancing stability and control during the throwing motion. Shot put throwing requires the use of key muscles; therefore, training plans should be customized for each individual based on their unique body type, muscle fiber composition, and neuromuscular coordination [5].

Height and BMI are two factors that can affect performance, but they are only one component of a complex equation [6]. A preliminary study revealed significant kinematic variability in throwing styles among elite and sub-elite athletes, emphasizing the need for specialized training methods [7,8]. The importance of kinematic analysis in understanding shot put technique efficiency and performance has been highlighted, demonstrating how kinematic analysis can enhance athlete training and optimize performance strategies [9]. The exploration of the biological aspects of throwing performance in track and field sports, focusing on the neurological and muscular components that influence athlete performance, contributed to the development of more effective training regimens [10].

The summary of biomechanical studies on shoulder kinematics in overhead sports identified research gaps and trends, serving as a guide for future studies on shoulder dynamics [11]. The focus on the validity of assessments for evaluating athletes’ overhead throwing abilities underscored the importance of accurate assessment tools for monitoring athlete progress and preventing injuries [12]. The investigation of the predictive value of kinematic variables on shot put outcomes and novice athlete selection found significant correlations between specific kinematic factors and shot put accuracy, providing valuable guidance for athlete selection and training [13].

The examination of the relationship between upper-limb function, shoulder mobility, and trunk strength in overhead athletes with shoulder pain emphasized the importance of comprehensive musculoskeletal evaluations for pain management and performance enhancement [14]. The study protocol presented for evaluating the effects of customized, pleasure-oriented exercise sessions in health clubs investigated how exercise adaptation impacts participant satisfaction and motivation [15].

Need for This Study: A thorough kinematic chain workout regimen can enhance throwing ability and shoulder muscular strength in collegiate players, according to research on shot put performance. However, the precise effect of these workouts on performance parameters like throwing velocity and distance is not well understood. To address this gap, a focused randomized control trial is required to determine the specific impact of kinematic chain exercises on shot put athletes. This research represents a novel approach to shot put training, as it uniquely focuses on quantifying the direct effects of kinematic chain exercises on key performance metrics. By exploring these specific impacts, our study will add to the existing body of knowledge on shot put training techniques, providing valuable insights into performance optimization and injury prevention that have not been previously addressed.

This study aims to evaluate the effectiveness of integrated kinematic chain exercises on shoulder muscle strength and throwing performance in collegiate shot put athletes compared to conventional training methods.

Hypothesis: We hypothesize that a kinematic chain-focused exercise regimen will significantly enhance shoulder muscle strength and throwing distance in collegiate shot put athletes compared to traditional training approaches.

## 2. Materials and Methods

### 2.1. Study Design and Setting

**Figure 1:**
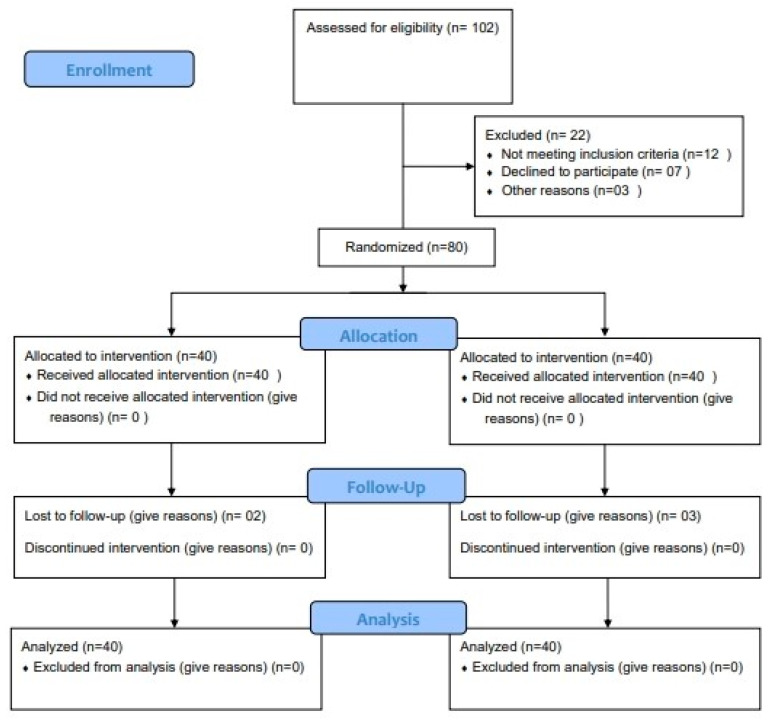
Flow of the study design.

This research was carried out at Dayananda Sagar University in Karnataka, India, between August 2022 and March 2024. It was planned as a randomized, controlled experiment. On 4 August 2023, the College of Physiotherapy’s Institutional Ethics Committee (IEC/IRB/DSU/FAC/2023/001) obtained approval for this experiment. Before this study began, the protocol was registered at the Clinical Trials Registry of India (CTRI/202309057242). The CONSORT (reporting a randomized trial) checklist from 2010 was followed in the reporting of this randomized controlled experiment [16]. There were two groups in this study: the experimental group (EG) and the control group (CG). Participants were assigned at random to either the intervention or control groups. To minimize bias in group allocation and maintain blinding, allocation concealment procedures were employed [17]. Figure 1 illustrates the study design’s flow.

### 2.2. Sample Size Calculation

A power analysis performed with G*POWER 3.1 determined that eighty participants were required for the current study to achieve a power of 0.80, an effect size of 0.75, and an alpha level of 0.05. The allocation ratio was 1:1. These parameters were chosen to ensure the study had sufficient statistical power to detect significant effects and was based on a combination of statistical criteria, previous research findings, and practical considerations related to participant recruitment [18]. These parameters ensured the study had sufficient statistical power to detect significant effects while being grounded in the context of the relevant literature and accessible sample size. All subjects provided informed, written consent before participation.

### 2.3. Participants

Posters and fliers were given to potential participants in sports centers in Bengaluru and surrounding areas. Also, local social media channels, such as Facebook groups and WhatsApp, were used to reach them. Shot put athletes were included in this study if they met the following inclusion criteria: healthy shot put athletes with a 2021 PAR-Q+ pre-participation evaluation questionnaire score of zero and a Disabilities of the Arm, Shoulder, and Hand (DASH)—Sports/Performing Arts Module score of 0%. Both male and female shot put athletes aged between 19 and 24 years old were eligible, provided they achieved a minimum shot put throwing distance from the power position of 8 m and above for males using a 6 kg shot put and 6 m and above for females using a 4 kg shot put. Furthermore, athletes had to reside in and around Bangalore, sign the consent form, and strictly follow the exercise protocol in order to participate in the study. The study group consisted of 80 shot put athletes who met these criteria, ensuring a homogeneous and well-defined sample for the research. Subjects were excluded from the study if they reported a history of shoulder, waist, or chest surgery; a fracture or dislocation of the affected shoulder; inflammatory joint disease; fibromyalgia; elbow injuries; wrist and hand injuries; shoulder trauma or current shoulder pathology (such as traumatic injury, glenohumeral joint dislocation, or acromioclavicular joint separation); or a history of recent upper-limb trauma. Additionally, subjects with a history of recent trauma to the lower limbs or surgery involving the hip, knee, or ankle; hip trauma or present hip, knee, and ankle pathology; or a recent history of spinal trauma, including lumbar, thoracic, and cervical trauma, or any ongoing spinal pathology (such as intervertebral disc prolapse, lumbar spondylosis, or cervical spondylitis) were also excluded. Athletes with severe musculoskeletal conditions, such as osteoarthritis, that impaired their ability to perform exercises were not included in the study [19].

### 2.4. Procedure

Two consecutive days of evaluation sessions made up this randomized controlled experiment. University shot put participants were examined for inclusion and exclusion criteria on the first day. Following that, participants completed the consent form and affirmed their desire to participate in the research. Following a two-day period, the subjects underwent another evaluation to determine baseline data, including height, weight, BMI, preferred throwing style, shot put throwing distance, and shoulder muscle strength measured using a hand-held dynamometer (HHD).

Randomizing and blinding:

We employed steps to achieve effective concealment allocation. First, a random allocation sequence was generated using a computer-based random number generator. This sequence determined the assignment of participants to either the intervention group or the control group, maintaining a 1:1 allocation ratio. The random allocation sequence was securely stored and not accessible to the researchers who enrolled participants. This was achieved by using opaque, sealed envelopes or a centralized randomization service. By following these procedures, we ensured that participants’ allocation to the intervention or control group was unbiased and concealed from both participants and the outcome assessor involved in the study [20].

In the sealed envelopes method, each participant’s allocation was placed in an opaque envelope, sealed, and numbered sequentially according to the random allocation sequence. These envelopes were prepared and secured by an independent third party who was not involved in the enrollment or assessment of participants. When a participant was deemed eligible and consented to participate, they were assigned a sequential number. The corresponding envelope was then opened by a designated individual who was not involved in the study’s assessment or intervention delivery. This ensured that the allocation sequence remained concealed until the participant was assigned to a group.

### 2.5. Outcome Measures

#### 2.5.1. Baseline Characteristics

The following baseline characteristics were collected: age, height, and weight

##### BMI Measurement

Body Mass Index (BMI) is a person’s weight in kilograms divided by the square of their height in meters. To measure an individual’s weight, we obtained their weight in kilograms (kg) using a calibrated scale, ensuring they were wearing minimal clothing and no shoes, and recorded the weight measurement to the nearest 0.1 kg. To measure the individual’s height in meters (m), we used a stadiometer or a wall-mounted height scale. The individual stood upright with their back against the wall, heels together, and head in the Frankfort horizontal plane, and the height measurement was recorded to the nearest 0.01 m. To calculate BMI, we squared the individual’s height measurement in meters (m^2^), and then divided their weight in kilograms (kg) by the squared height (m^2^). The formula for calculating BMI was as follows: weight (kg)/height (m^2^) [21,22].

*Shot Put Throwing Distances*

The International Association of Athletics Federations (IAAF) set official rules for the assessment of shot put throwing distance. These rules ensured accuracy and fairness in the sport. Participants had to meet weight and size specifications, use a calibrated tape measure, and accurately measure the distance. The throwing sector was marked with lines at 34.92 degrees from the center of the circle, and the area was cleared of obstacles. Participants performed a warm-up routine and wore appropriate clothing and footwear. The athlete entered the circle from the back without touching the top of the stop board or circle’s rim, positioned the shot close to the neck, and exited the circle from the back half. The distance was recorded to the nearest centimeter [23].

##### Assessment of Muscle Strength Using a Dynamometer

A dynamometer [JTECH Commander Echo Muscle Tester (JTECH Medical, Salt Lake City, UT, USA)] hand-held dynamometer] was used to assess key muscle strength in the shoulder. The hand-held dynamometer was calibrated and functioning correctly before use, and the test was conducted in a quiet, comfortable setting to minimize distractions and enhance concentration. Participants were instructed to perform a maximum voluntary contraction (MVC) against the dynamometer, with adjustments made for optimal results and participant comfort. The dynamometer measured the maximum force exerted in units of kilograms during the test, which was repeated three times. The highest value was recorded, providing sufficient rest between trials to prevent muscle fatigue. The maximum force values obtained were documented.

To measure shoulder flexion strength, participants were seated comfortably with their backs supported, and their arms flexed forward to approximately 90 degrees. The dynamometer pad was placed on the anterior aspect of the arm just above the wrist, ensuring it was perpendicular to the arm’s movement axis for accurate force readings. The dynamometer displayed the force generated by the participant’s shoulder extension, and the measurement was recorded.

For measuring extension strength, the participant sat upright with their shoulder in a neutral position. The examiner stabilized the shoulder, and a handheld dynamometer was placed on the upper arm near the triceps muscle. The participant extended their arm against the dynamometer, applying counter pressure. The force generated was recorded, and the highest value was taken as the final measurement.

To measure shoulder internal rotation strength, the shoulder was kept neutral, and the elbow was positioned at 90 degrees flexion. The dynamometer was placed at the wrist area to measure the internal rotation motion. The participant rotated the arm inward against the dynamometer’s resistance, and the highest value was recorded as the final measurement.

To measure shoulder external rotation strength, the participant’s shoulder was tested using a dynamometer, positioned in a neutral position. The examiner stabilized the shoulder to prevent compensatory movements. The dynamometer was placed against the dorsal aspect of the participant’s forearm near the wrist. The participant externally rotated their shoulder against the resistance provided by the dynamometer, and the highest value or average of three attempts was recorded as the final measurement [24].

### 2.6. Intervention

Intervention integrates exercises targeting the lower body, core, and upper body to enhance muscle strength and coordination, ultimately aiming to improve shot put performance [19,25]. Detailed descriptions of the exercise protocols used in this study are provided in Table 1.

### 2.7. Data Analysis

The statistical software SPSS version 28.0 was used for the analyses. The minimum, maximum, mean values and standard deviation of the baseline clinical characteristics of the participants were calculated and reported with *p* values. The normality of the data was evaluated using various graphical and formal statistical methods, including histograms, Q-Q plots, z-scores of kurtosis and skewness, and lastly, the Shapiro-Wilk test. Two-way ANOVA for in-group analysis was used to find the change in shot put throwing distance and muscle strength. Lastly, associations between height and BMI (dependent variables) and shot put throwing distance (independent variables) were analyzed using Pearson’s correlation coefficient tests. *p*-values < 0.05 were considered statistically significant.

## 3. Results

Eighty participants (male, fifty, and female, thirty) completed this study. We conducted detailed analysis and insights into the mean, standard deviation, and statistical significance (*p*-values) of each variable at the start of the study, allowing for a comprehensive understanding of the differences between the control and experimental groups and the homogeneity between the groups. Age and gender, the baseline characteristics of the participants, are shown in Table 1.

Table 2 presents the frequencies of gender distribution and preferred throwing style among participants, divided into control and experimental groups. The gender distribution is evenly split, with both the control and experimental groups each comprising 25 males (31.3% of the total participants) and 15 females (18.8% of the total participants). This results in a balanced gender representation, with 62.5% of participants being male and 37.5% female. Such a distribution helps ensure that gender-related biases are minimized in the study’s outcomes.

Regarding preferred throwing styles, the data shows that 48.8% of participants prefer the gliding style, with 20 individuals in the control group and 19 in the experimental group. Conversely, 51.3% prefer the rotational style, with 20 participants in the control group and 21 in the experimental group. This indicates a slight overall preference for the rotational style, especially within the experimental group. The even distribution of throwing styles across the groups provides a comprehensive understanding of participants’ baseline characteristics, which is crucial for interpreting the study results and potential interactions between throwing styles and interventions.

Table 3 outlines the baseline characteristics of the randomized study participants, providing a detailed comparison between the control and experimental groups across various measurements.

For age, the control group had a mean age of 20.74 years (SD = 1.41), while the experimental group was slightly younger, with a mean age of 19.84 years (SD = 1.54). The difference between groups was statistically significant, with a *p*-value of 0.008, indicating that age varied between the groups at baseline.

In terms of height, both groups were almost identical, with the control group averaging 166.06 cm (SD = 4.14) and the experimental group averaging 166.03 cm (SD = 4.41). The *p*-value of 0.979 suggests no significant difference in height between the groups.

Weight measurements revealed a mean weight of 68.4 kg (SD = 10.00) for the control group and 69.41 kg (SD = 9.77) for the experimental group, with a *p*-value of 0.649, indicating no significant difference in weight.

For Body Mass Index (BMI), the control group had a mean BMI of 24.65 kg/m^2^ (SD = 2.71), while the experimental group had a mean BMI of 25.02 kg/m^2^ (SD = 2.55). The *p*-value of 0.538 shows no significant difference in BMI between the two groups.

When examining pre-throwing distance, the control group had a mean of 8.38 m (SD = 1.16) compared to 8.28 m (SD = 1.13) for the experimental group, with a *p*-value of 0.69, indicating no significant difference.

Regarding pre-flexor muscle strength, the control group had a mean strength of 23.15 kg (SD = 5.62), while the experimental group had a mean of 25.2 kg (SD = 6.49). The *p*-value of 0.135 suggests a marginal difference in flexor muscle strength between the groups, though it is not statistically significant.

For pre-extensor muscle strength, the control group had a mean of 15.43 kg (SD = 3.13), and the experimental group had a mean of 14.4 kg (SD = 2.07). The *p*-value of 0.088 indicates a potential trend towards a difference in extensor strength, but it is not statistically significant.

Pre-internal rotator muscle strength showed the control group with a mean of 11.36 kg (SD = 1.98) and the experimental group with a mean of 11.11 kg (SD = 1.80), with a *p*-value of 0.557, suggesting no significant difference.

Lastly, for pre-external rotator muscle strength, the control group averaged 10.74 kg (SD = 2.37), and the experimental group averaged 11.39 kg (SD = 1.99), with a *p*-value of 0.187, indicating no significant difference between the groups.

The two-way ANOVA is a statistical test used to analyze the effects of exercise protocols on the performance and strength of shot put athletes. This test examines the interaction between different exercise protocols and their effects on the dependent variables. By comparing the means of multiple groups, the test helps determine if the observed differences are statistically significant (Table 4).

**Figure 2:**
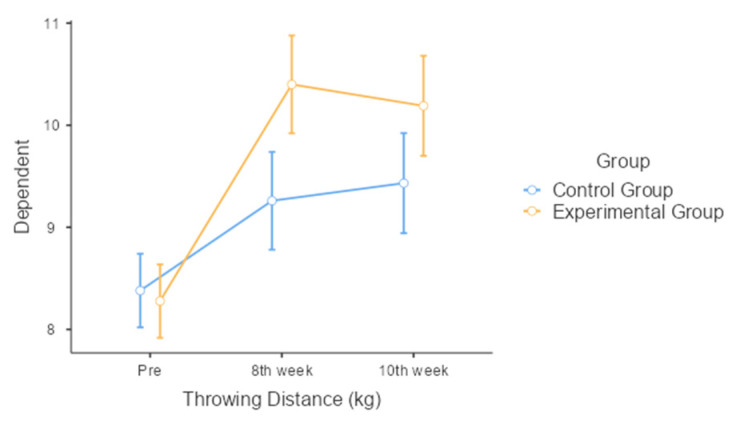
Comparison of throwing distance over time between control and experimental groups.

Throwing Distance: The results show that both the control and experimental groups experienced significant improvements in throwing distance over the course of the intervention period. Initially, the control group had an average throwing distance of 8.38 m, which increased to 9.26 m at the 8th week and further to 9.43 m at the 10th week. Similarly, the experimental group started with an average distance of 8.28 m, improving significantly to 10.4 m by the 8th week and slightly decreasing to 10.19 m by the 10th week. While both groups improved, the experimental group exhibited a greater increase in throwing distance, with significant improvements noted at both the 8th and 10th weeks compared to the control group. Despite this, the interaction effect between group and time on throwing distance was not statistically significant (*p* = 0.053), although it was close to the threshold, indicating a trend towards greater improvement in the experimental group (Figure 2).

Shoulder Muscle Strength: The findings on shoulder muscle strength revealed notable differences between the control and experimental groups across various muscle groups.

**Figure 3:**
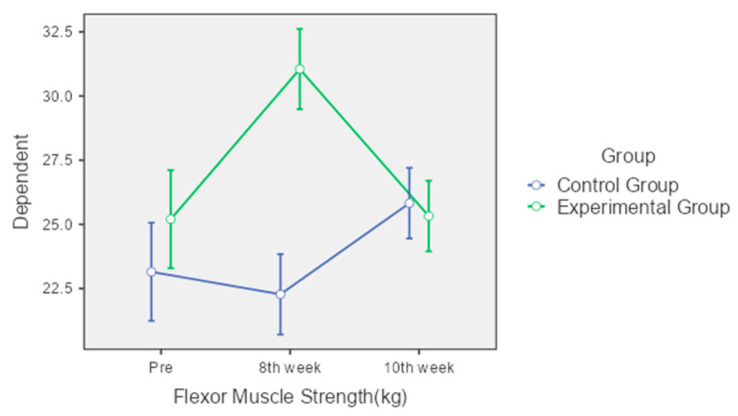
Comparison of flexor muscle strength over time between control and experimental groups.

Flexor Muscle Strength: The control group’s flexor muscle strength showed no significant change from pre-intervention to the 8th week but increased significantly by the 10th week (from 23.15 kg to 25.82 kg). In contrast, the experimental group exhibited a significant increase from pre-intervention to the 8th week (from 25.2 kg to 31.05 kg), maintaining a significant improvement over the control group at this point. However, by the 10th week, the experimental group’s flexor muscle strength had returned to levels similar to the pre-intervention period (Figure 3).

**Figure 4:**
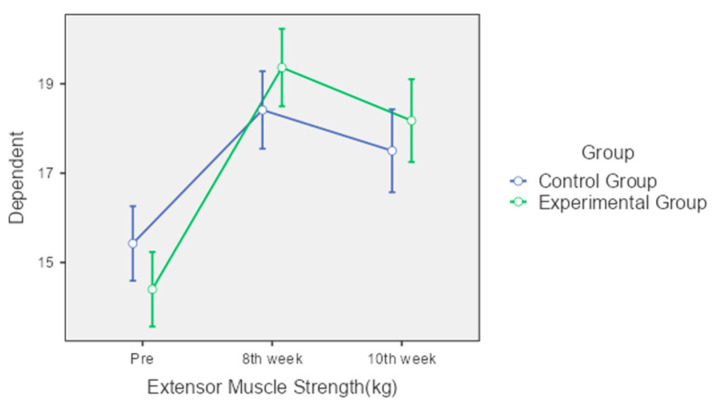
Comparison of extensor muscle strength over time between control and experimental groups.

Extensor Muscle Strength: The control group demonstrated significant improvements in extensor muscle strength at both the 8th and 10th weeks (from 15.43 kg to 18.41 kg and 17.5 kg, respectively). The experimental group also showed significant increases, from 14.4 kg at pre-intervention to 19.36 kg at the 8th week and 18.18 kg at the 10th week. Although both groups improved, the experimental group showed slightly better performance, but the interaction effect was not statistically significant (*p* = 0.73) (Figure 4).

**Figure 5:**
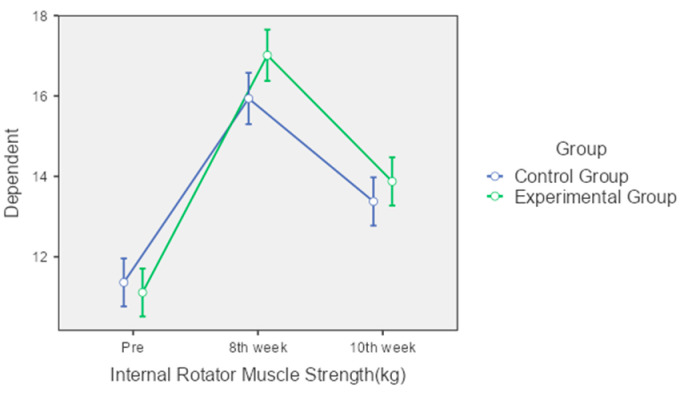
Comparison of internal rotator muscle strength over time between control and experimental groups.

Internal Rotator Muscle Strength: Both groups experienced significant increases in internal rotator muscle strength. The control group improved from 11.36 kg to 15.94 kg at the 8th week and slightly decreased to 13.38 kg by the 10th week. The experimental group showed a more pronounced improvement, from 11.11 kg to 17.01 kg at the 8th week, though this declined to 13.88 kg by the 10th week. Despite these improvements, the interaction effect between group and time was not statistically significant (*p* = 0.302) (Figure 5).

**Figure 6:**
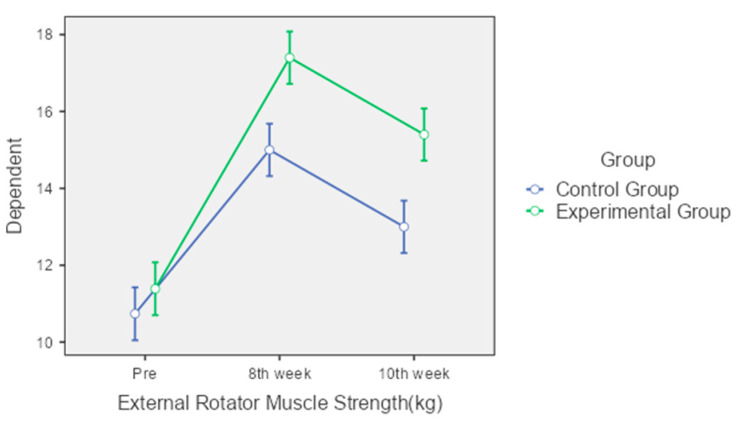
Comparison of external rotator muscle strength over time between control and experimental groups.

External Rotator Muscle Strength: The control group’s external rotator muscle strength significantly increased from 10.74 kg to 15 kg at the 8th week and slightly decreased to 13 kg by the 10th week. The experimental group showed significant and consistent improvements from 11.39 kg at pre-intervention to 17.4 kg at the 8th week and 15.4 kg at the 10th week. The interaction effect for external rotator muscle strength was statistically significant (*p* < 0.001), highlighting the superior performance of the experimental group over time (Figure 6).

The study examined correlations between height, BMI, and throwing distance among shot put throwers. The results showed a strong positive correlation between height and throwing distance, with a Pearson correlation coefficient of 0.8055. On average, athletes had a height of 1.6705 m (SD = 0.042) and threw a distance of 8.55 m (SD = 1.14) (Table 5).

## 4. Discussion

This study provides a comprehensive analysis of the physical characteristics and performance measures of participants, offering detailed insights into the mean, standard deviation, and statistical significance of various variables. The initial assessment of age and gender distributions between the control and experimental groups demonstrated no significant differences, ensuring homogeneity between the groups at the beginning of the study. The balanced representation of both males and females in the groups further strengthened the baseline equivalency, laying a solid foundation for the comparative analysis.

This study also meticulously recorded pre-intervention measures of throwing distance and shoulder muscle strength, encompassing flexion, extension, internal rotation, and external rotation. These baseline performance measures were fundamental in assessing the efficacy of the intervention. By establishing these metrics before the intervention, this study ensured that any improvements observed could be attributed to the experimental procedure rather than natural progression or training outside the study parameters.

Throwing Distance

This study’s findings indicate a significant increase in throwing distance for the experimental group (EG) compared to the control group (CG). This outcome could potentially be attributed to the kinematic chain activation used in the EG, which might have been more successful in improving the muscle strength and coordination needed for throwing-related activities. According to research by Cools et al. [26], customized sport-specific rehabilitation programs can greatly impact throwing ability. The considerable difference in throwing distance between the EG and the CG among athletes highlights the efficacy of the experimental procedure designed for the athletes; this is consistent with research by Vrublevskiy et al. [27], which indicated that male and female patients receiving shoulder rehabilitation exhibit different adaptation and improvement patterns.

Shoulder Muscle Strength: Flexion (FLX) and External Rotation (ER)

The EG’s FLX and ER mean values were significantly higher, indicating that the experimental exercises were beneficial to these particular motion patterns. This improvement could be attributed to the targeted strengthening of the external rotator muscles and posterior chain. The significant improvement in FLX among athletes is particularly noteworthy because it affects throwing mechanics and shoulder stability. Researchers Wight et al. [28] discovered that athletes with shoulder injuries might achieve markedly better results with customized rehabilitation regimens that prioritize ER strength.

Extension (EXT) and Internal Rotation (IR)

The EG’s EXT and IR mean values were not significant, indicating that among male subjects, the experimental technique may not have been as effective at enhancing the strength of those movements as it was for other motions. This could be a result of the specific exercises chosen for the EG, which may have highlighted different facets of shoulder functionality. The multimodal approach required for thorough shoulder rehabilitation is discussed in a paper by Ellen Becker, Todd S. Aoki, and Ryoki [29], emphasizing the significance of focusing on different muscle groups for overall shoulder strength and stability.

Correlation between Height, BMI, and Throwing Distance

The correlation coefficient (r), which is close to 1, suggests a strong positive linear relationship between height and throwing distance, as well as between BMI and throwing distance. A correlation coefficient close to 1 indicates a strong tendency for the variables to move in the same direction; in this case, as height or BMI increases, the throwing distance also tends to increase. This finding implies that taller athletes and those with higher BMI measurements generally achieve greater shot put throwing distances. The finding was in line with a study by Aikawa et al. [30]; taller athletes likely benefit from longer lever arms and increased muscle mass, providing them with more power and momentum to propel the shot put further. Similarly, athletes with a higher BMI might possess greater muscle strength and mass, contributing to their ability to throw the shot over longer distances.

## 5. Conclusions

This study’s findings indicate that the experimental training regimen significantly improved the subjects’ throwing distance, key shoulder muscle strength attributes, particularly among females, and internal rotation and extension measurements. This emphasizes the significance of developing tailored, gender-specific rehabilitation plans that consider each participant’s specific needs and physiological traits. Further research should examine the particular components of the experimental procedure that most significantly influenced these results, with an emphasis on optimizing rehabilitative tasks to achieve specific improvements in patient satisfaction and shoulder function.

Limitations: It is critical to recognize some of our study’s shortcomings. The duration of the follow-up period was somewhat brief, which naturally corresponded with the length of time that athletes received free training. Furthermore, the study was limited to a single center due to logistical difficulties, which may have limited the overall applicability of the findings.

## Figures and Tables

**Table 1:** Description of exercise protocol.

Conventional Training Protocol			Kinematic Chain Exercise Protocol	
Weeks	Phase	Description	Phase	Description
Weeks 1–2:	Foundation phase: Focus on building a solid foundation of strength and movement patterns. Strength Training:	Increase intensity by lifting weights. Aim for 3–4 sets of 4–8 reps. Focus on exercises like squats, lunges, Romanian deadlifts, bench presses, and pull-ups. Power Development: Include more advanced power exercises such as barbell cleans, snatch variations, and plyometric exercises like depth jumps and explosive pushups. Shot Put Technique: Continue practicing shot put technique drills.	Foundation phase: Focus on building a solid foundation of strength and movement patterns.	Squat Variations: Perform barbell squats, goblet squats, and Bulgarian split squats to strengthen the lower body. Aim for 3–4 sets of 4–8 reps. Medicine Ball Throws: Include exercises like overhead medicine ball slams, rotational throws, and chest passes to develop power and explosive strength. Core Stability: Incorporate exercises like planks, side planks, and Russian twists to develop core stability and rotational strength.
Weeks 3–4:	Strength and power focus: Continue to build strength while increasing power output. Strength Training:	Maintain strength gains by continuing with heavy compound exercises. Aim for 3–4 sets of 6–10 reps. Power Development: Emphasize explosive movements such as power cleans, snatches, and jump squats. Shot Put Technique: Allocate more time for technical skill work. Practice shot put throws with lighter implements to refine release technique and improve speed of movement.	Strength and power focus: Continue to build strength while increasing power output.	Deadlift Variations: Include exercises like conventional deadlifts, trap bar deadlifts, and Romanian deadlifts to strengthen the posterior chain. Aim for 3–4 sets of 6–10 reps. Kettlebell Swings: Perform kettlebell swings to develop explosive hip power. Plyometric Exercises: Incorporate box jumps, depth jumps, and bounding to improve power output.
Weeks 5–6:	Power and explosiveness: Shift the focus towards explosive power and increased speed. Strength Maintenance:	Maintain strength gains by reducing volume but maintaining intensity. Aim for 2–3 sets of 8–12 reps. Power Maintenance: Continue with explosive exercises, but reduce volume. Technique: Focus on fine-tuning technique, performing shot put throws with competition weight implements. Incorporate competition-specific scenarios and mental preparation.	Dynamic movements and stability	Lunge Variations: Include walking, reverse, and lateral lunges to improve lower body strength and stability. Aim for 3–4 sets of 8–12 reps. Medicine Ball Rotational Throws: Perform exercises like rotational shot put throws with a medicine ball to enhance power and rotational strength. Single-Leg Balance Exercises: Incorporate single-leg deadlifts, single-leg squats, and pistol squats to improve stability and balance.
Weeks 7–8:	Peaking phase: Taper the training to optimize performance for competitions.	Throughout the entire 8-week protocol, it is important to prioritize proper warmups, cooldowns, and recovery strategies such as stretching, foam rolling, and adequate rest.	Integration and peaking phase. Cleans and Snatches:	Include barbell cleans and snatches to develop explosive power and coordination throughout the kinematic chain. Aim for 3–4 sets of 8–12 reps. Overhead Press Variations: Perform exercises like overhead barbell presses, dumbbell presses, and push presses to strengthen the upper body. Shot Put-Specific Drills: Allocate more time for practicing shot put throws with competition weight implements. Focus on technique, coordination, and timing.

**Table 2:** Frequencies of gender and preferred throwing style.

Gender	Group	Counts	% of Total	Cumulative %
Male	Control Group	25	31.3 %	31.3 %
	Experimental Group	25	31.3 %	62.5 %
Female	Control Group	15	18.8 %	81.3 %
	Experimental Group	15	18.8 %	100.0 %
Preferred Throwing Style	Group	Counts	% of Total	Cumulative %
Gliding Style	Control Group	20	25.0 %	25.0 %
	Experimental Group	19	23.8 %	48.8 %
Rotational Style	Control Group	20	25.0 %	73.8 %
	Experimental Group	21	26.3 %	100.0 %

**Table 3:** Mean standard deviation and *p* value for baseline data. Baseline characteristics of the randomized study participants (*N = 80*).

	Group	N	Mean	SD	*p*
Age (Years)	Control Group	40	20.74	1.408	0.008
	Experimental Group	40	19.84	1.536	
Height (Cms)	Control Group	40	166.06	4.137	0.979
	Experimental Group	40	166.03	4.411	
Weight (Kg)	Control Group	40	68.4	9.997	0.649
	Experimental Group	40	69.41	9.766	
BMI (Kg/m^2^)	Control Group	40	24.65	2.71	0.538
	Experimental Group	40	25.02	2.545	
Pre-Throwing Distance	Control Group	40	8.38	1.159	0.69
	Experimental Group	40	8.28	1.128	
Pre-Flexor Muscle Strength	Control Group	40	23.15	5.618	0.135
	Experimental Group	40	25.2	6.485	
Pre-Extensor Muscle Strength	Control Group	40	15.43	3.125	0.088
	Experimental Group	40	14.4	2.07	
Pre-Internal Rotator Muscle Strength	Control Group	40	11.36	1.984	0.557
	Experimental Group	40	11.11	1.799	
Pre-External Rotator Muscle Strength	Control Group	40	10.74	2.367	0.187
	Experimental Group	40	11.39	1.989	

Abbreviations: ER, external rotation; IR, internal rotation; FLX, flexion; EXT, extension: mts, meters; kg, kilogram; BMI, Body Mass Index.; %, percentage; Cms, centimeters. Continuous data are expressed as mean (standard deviation), categorical data as number, and *p* values are associated with two-sample *t*-tests, which are used for continuous variables. SD, Standard Deviation, *p* = 0.05 significance level.

**Table 4:** Comparisons of outcome between groups in throwing distance and muscle strength at pre-, 8th, and 10th week.

Parameters	Control Group (n = 40)			Experimental Group (n = 40)			Group X Time ^#^
	Pre-	8th Week	10th Week	Pre-	8th Week	10th Week	*p*
Throwing Distance (mts)	8.38 ± 1.16	9.26 ± 1.13 ^+^	9.43 ± 4.21 ^+^	8.28 ± 1.13	10.4 ± 1.46 ^+^	10.19 ± 1.58 ^+^*	0.053
Shoulder Muscle Strength (kg)							
Flexor Muscle Strength	23.15 ± 5.62	22.27 ± 4.59	25.82 ± 4.59 ^+^	25.2 ± 6.49	31.05 ± 5.33 ^+^*	25.32 ± 0.4.52	< 0.001
Extensor Muscle Strength	15.43 ± 3.12	18.41 ± 3.31 ^+^	17.5 ± 3.36 ^+^	14.4 ± 2.07	19.36 ± 2.06 ^+^	18.18 ± 2.46 ^+^*	0.73
Internal Rotator Muscle Strength	11.36 ± 1.98	15.94 ± 2.12 ^+^	13.38 ± 1.97 ^+^	11.11 ± 1.8	17.01 ± 1.93 ^+^*	13.88 ± 1.85 ^+^	0.302
External Rotator Muscle Strength	10.74 ± 2.37	15 ± 2.35 ^+^	13 ± 2.35 ^+^	11.39 ± 1.99	17.4 ± 1.96 ^+^*	15.4 ± 1.96 ^+^*	< 0.001

All data are expressed as means ± SD; ^#^ = Analysis of two-way repeated measures ANOVA; * = significant difference between groups at *p* < 0.05; ^+^ = significant difference pre- and post-intervention at *p* < 0.05.

**Table 5:** Correlations between height, BMI, and throwing distance in male and female shot put thrower athletes.

Correlations between Height and Throwing Distance:			
Sample size	Height (mts) Means and SD	Throwing Distance (mts) Means and SD	Pearson correlation coefficient (r)
n = 80	1.6705 ± 0.042	8.55 ± 1.14	0.8055
Correlations between BMI and Throwing Distance:			
	BMI (kg/mts^2^)	Throwing Distance (mts) Means and SD	Pearson correlation coefficient (r)
n = 80	26.09 ± 2.39	8.55 ± 1.14	0.7598

Similarly, the study found a significant positive correlation between BMI and throwing distance, with a Pearson correlation coefficient of 0.7598. The athletes had an average BMI of 26.09 kg/m^2^ (SD = 2.39) and maintained the same average throwing distance of 8.55 m (SD = 1.14). mts, meters; BMI, Body Mass Index; SD, standard deviation.

## Data Availability

The data that support the findings of this study are available upon request from the corresponding author, V.K.K.C.G.
